# A stacked custom convolution neural network for voxel-based human brain morphometry classification

**DOI:** 10.1038/s41598-025-17331-4

**Published:** 2025-10-02

**Authors:** T. Arumuga Maria Devi, K. S. Saji

**Affiliations:** Manonmaniam Sundaranar University, Tirunelveli, Tamilnadu India

**Keywords:** Brain tumor, Convolutional neural network, Classification, Segmentation, Voxel-based morphometry, Engineering, Biomedical engineering

## Abstract

The precise identification of brain tumors in people using automatic methods is still a problem. While several studies have been offered to identify brain tumors, very few of them take into account the method of voxel-based morphometry (VBM) during the classification phase. This research aims to address these limitations by improving edge detection and classification accuracy. The proposed work combines a stacked custom Convolutional Neural Network (CNN) and VBM. The classification of brain tumors is completed by this employment. Initially, the input brain images are normalized and segmented using VBM. A ten-fold cross validation was utilized to train as well as test the proposed model. Additionally, the dataset’s size is increased through data augmentation for more robust training. The proposed model performance is estimated by comparing with diverse existing methods. The receiver operating characteristics (ROC) curve with other parameters, including the F1 score as well as negative prediction value, are used to determine the proposed model effectiveness. The proposed VBM and stacked custom CNN model achieves significant improvements. The proposed model performed with a higher accuracy level of 98%. The results confirm that the proposed VBM and stacked custom CNN model outperforms other existing approaches in brain tumor classification.

## Introduction

The non-invasive imaging of the structure, function, or pharmacology of the nervous system is known as neuro imaging^1,2^. The most prevalent type of medical imaging at the moment is magnetic resonance imaging (MRI), which can clearly demonstrate the architecture of brain tissue without causing damage from ionizing radiation^3^. MRI is currently the least ordinary type of medical imaging because it can clearly show the architecture of brain tissue without causing damage from ionizing radiation^4,5^. As a result, a number of analyses^6^ effort to use MRI along with computer-aided technology to identify AD patients’ brain atrophy.

Indeed, handcrafted feature-rooted machine learning (ML) methods like logistic regression (LR), support vector machine (SVM) as well as random forest (RF) have dominated previous research on computer-aided schizophrenia classification using brain structural MRI^7,8^. The unreliability of selected features, combined with the subtle, sparse, and inconsistently distributed brain atrophy patterns in schizophrenia, limits the generalizability and performance of handcrafted-feature-based ML methods. As a result, the request of computer-aided analysis in the actual world is restricted^9^. CNN, a data-driven approach, can perform automatic feature learning, removing the need for human judgment when selecting relevant spatial features^10^.

The signal-to-noise ratio is a concern in MRI images, which can be improved through preprocessing. Numerous studies have used voxel-based morphometry (VBM) to analyze MRI^3^. Spatial smoothing is one of those VBM steps that uses gaussian smoothing (GS) to reduce noise. GS, alters the original images during denoising^11^. Hence, to work with the limitations like noise, segmentation and accurate classification this work’s gives the following contribution:

- The proposed approach combines median filter and segmentation, particularly canny edge detection, to normalize the input images before giving input into the proposed stacked custom CNN.
- For detecting brain tumors images, a stacked custom CNN network with nearly 15 layers is developed. A custom activation layer is also created.
- In addition, this work makes use of data augmentation, regularization, and batch normalization to boost the proposed stacked custom CNN model accuracy.

While existing works have independently explored VBM and CNNs, this study presents a specific contribution through the combination of a customized CNN architecture. Further, this work is embedded with the novel SPR-eLu activation function, optimized VBM-based preprocessing (adaptive median filtering + Canny edge detection), and a fully ablated evaluation across multiple data augmentations and loss behaviors. Unlike existing approaches, as in^1,12^, which generally employ standard ReLU-based CNNs with minimal morphological enhancement, the proposed approach systematically strengthens voxel-level structural texture before classification, enhancing segmentation accuracy and decision stability. The resulting model consistently outperforms comparable existing approaches in accuracy, convergence speed, and loss regularity, underscoring its effectiveness for morphometric classification in brain imaging.

### Clinical significance of brain tumor detection

Brain tumors accurate classification and early detection are important for prognosis assessment, treatment planning, and surgical decision-making. Brain tumors, including meningiomas, gliomas, and pituitary tumors, vary in their aggressiveness, growth rates, and imaging characteristics. Meningiomas appear as well-circumscribed, extra-axial masses, whereas Gliomas, are heterogeneous and infiltrative on MRI. To improve clinical outcomes, understanding these distinctions is vital. Automated detection systems assist radiologists and enhance clinical workflow efficiency by offering preliminary assessments, aiding early diagnosis, and reducing human error.

The remaining article are organized as follows: In the second part of our study, we evaluate the most recent existing works. Section Proposed methodology defines the proposed model, while Sect. Results discusses and presents its outcomes. In Sect. Conclusion, after the references, you will find the study conclusion and some suggestions for future paths.

## Literature review

This study provides an outline of some of the most recent researchers working on VBM and CNN-based brain image analysis.

Vyškovsky et al.^2^ utilized three-dimensional CNN and stacked auto encoders (SAE). The data has only been minimally preprocessed, and determine whether advanced feature extraction techniques can aid in increasing the DL-rooted classifiers accuracy. Three common preprocessing steps were used in the experiments to extract three distinct feature types. VBM, deformation-rooted straightforward along with morphometry spatial normalization of brain tissue were among them.

Dufumier et al.^12^ on both VBM pre-processed as well as minimally pre-processed quasi-raw images, proposed a benchmark of the latest state-of-the-art (SOTA) 3D CNN, assessing the advantages of deep ensemble learning as well as data augmentation. With VBM images, it is discovered that all models outperform quasi-raw data significantly in terms of prediction accuracy. As the training set methods 10,000 samples, where quasi-raw data nearly match VBM’s performance, this finding emerged. In addition, it was demonstrated that linear models make similarly to SOTA CNN on VBM data.

Levakov et al.^13^ presented a DL framework for predicting chronological age based on architecture MRI scans. Utilizing CNN analysis to locate brain part play a part in predicting the intricate, multivariable process of brain aging. “Explanation maps” were discovered when previous research looked into ways to concept pixel/voxel-wise contributions for single image prediction. To resolve this issue, this work fostered a surmising plan for joining the semaps across subjects.

Mendes et al.^14^ investigates structural MRI (sMRI) of the brain using data from two publicly available data sets—ABIDE-II as well as ADHD-200. To mental health, estimate age as well as gender position based on knowledge brain differences, a 3D CNN was trained with a multitask learning strategy using gray as well as white matter preprocessed via VBM. Attention maps were created using gradient-based techniques, which allowed for the clinically related finding of the most representative brain part for model decision-making. Predictions for gender and age that were satisfactory were made using this method.

Morita et al.^15^ highlighted that in order to detect developmental disorders very early in the neonatal period, a system based on brain shape analysis is needed. However, due to the wide mixture of brain shape deformations caused by developmental disorders, neonatal brain shape evaluation along with brain development is hard. To construct a statistical model for evaluating differences in the shape of the neonatal brain, a substantial dataset of the infant’s brain and their brain part mask are needed. The task of segmenting the brain takes a lot of time. As a result, the method for segmenting the brain of a newborn using CNN and fine-tuning it using adult brain datasets is presented in this paper.

Estudillo-Romero et al.^16^ gave a work that is frequently carried out in a whole-brain voxel-wise way. In this method, a statistical exam on the arrangement of image intensities for all placement is carried out on a population of healthy control and patient that are registered to a public coordinate space. Despite the fact that this approach has provided a count of scientific insights, it is further from clinical application due to the fact that the variation is frequently insignificant and do not permit a successful classifier.

Zhang et al.^17^ gathered 60 conduct disorder (CD) along with 60 age- as well as a gender-matched healthy controls (HCs) for high-resolution sMRI. This model was tested as well as trained using the five-fold cross validation (CV) system. This model’s and the SVM model’s receiver operating characteristic (ROC) curves were compared. A feature visualization was used to get a sense of the sMRI features that the Alex Net model learned. In sMRI of CD, parietal lobe, occipital lobe, the frontal lobe as well as superior temporal gyrus were highlighted by the saliency maps for all convolutional layer. The classification results showed that a DL-based method can find hidden CD features in sMRI images, which could help doctors diagnose CD.

Solana-Lavalle et al.^18^ carried out quantitative and qualitative analyses of MRI scans in order to better understand Parkinson’s disease. The use of VBM on an MRI to identify the gray matter part of interest where dopamine-producing nerve cells have been lost is the basis for some quantitative analyses. This study introduces a method for classifying a person’s 3-D magnetic resonance scans as an aid in the Parkinson’s disease diagnosis. Since gender shows an important role in Neuro biology, distinct studies are showed for women as well as men. Ueda et al.^19^ observed a morphological change in the human brain pattern throughout the development of the brain along with healthy aging. These patterns can be used to estimate the subjects’ ages from brain images. This paper proposes an age estimation technique rooted on brain T1-weighted images and 3-Dimensional CNN (3DCNN).

Sujatha et al.^20^ utilized the information from MRI in addition to the RF algorithm. These irregularities are useful for distinguishing bipolar disorder patients from mental health issues or health controls, which define illness based on their patients. ML algorithms were employed for accuracy, like CNN-MDRP (multimodal disease risk prediction) and the RF algorithm. The output is obtained by utilizing the trained ML algorithm and data set. The obtained MRI data will be divided and pre-processed using VBM. To understand the deviations in Gray Matter (GM) as well as White Matter (WM) of the various data groups individually, a straightforward equation is used. Additionally, the Principal Component Analysis will be employed. Recent developments in medical imaging have leveraged context-aware and transformer-based models such as U-KAN, P2SAM, GTP-4O, and others^21–28^. This highlighted the growing importance of modality-aware feature learning and ambiguous object segmentation. In future this work, will integrate such advanced models to further enhance voxel-based analysis.

## Proposed methodology

**Fig. 1:**
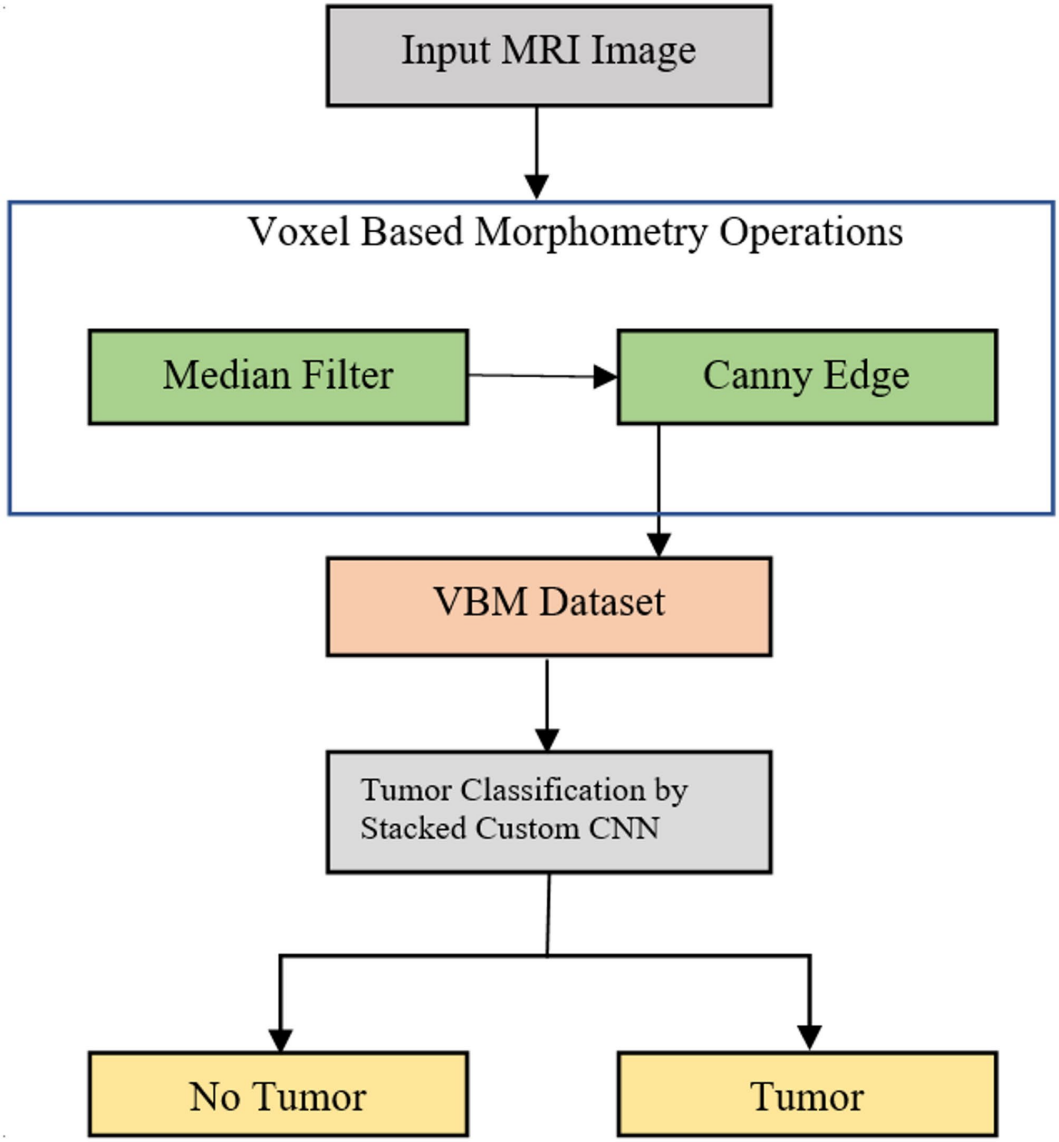
Proposed VBM Architecture with stacked custom CNN.

VBM operations follow the initial collection of the input dataset on brain tumors. In order to pre-process the dataset, the VBM operation includes adaptive median filtering. As a result, canny edge detection is used to perform a second VBM operation known as segmentation. The process of segmentation is simple. Segmenting and spatially normalizing high-resolution images are part of it. Therefore, the grey matter and white matter of the image are taken into consideration when segmenting the image. Finally, these VBM images are used for classification. A stacked custom DL strategy is used to classify the data. When compared to the standard DL algorithm, the stacked custom CNN model incorporates some enhancements. Figure 1 depicts the proposed VBM-based stacked custom CNN architecture.

### Augmentation of data

Consistency in the data hinders network convergence and makes it harder for the network for pattern matching. All of the images are uploaded to the data augmentation module in order to resolve this issue^21^. The images’ rotation, height, width, shear, zoom, and horizontal flipping are all taken into account for data augmentation. In the initial stage, this data augmentation resulted in the conversion of every MRI image dataset. Additionally, each image is resized to 240 × 320 pixels. The zoom operation uses a spline interpolation procedure of 0th order to replace the missing data to avoid over-interpolation. The following augmentation techniques are applied. Rotation makes random rotations between ± 15 degrees, by shear transformation, the shear intensity is within 0.2. With zoom range a random zoom is between 80 and 120% and 50% probability with horizontal flipping. These augmentations help in balancing the dataset and enhancing model generalization by introducing variability during training. The validation accuracy for the models trained with augmentation shows a 3–5% improvement when compared to models without augmentation. Following that, voxel-based morphology operations and a filter are applied to this image dataset. Subsequently, the result is given to the proposed stacked custom CNN.

### Voxel based morphology operation

When applied to MR images, VBM is utilized to extract specific part of interest in WM and GM. Both the WM and the GM in the filtered images are segmented. Differences between the two tumor groups are identified and localized through voxel-wise analysis. Medical images are frequently noisy, which significantly affects the segmentation of lesions and the diagnosis of patients. Hence, a filter is utilized to take away additional noise from the MRI dataset. Here, the adaptive median filter is utilized to remove noise from the images while keeping the edges. The median filter is given to all pixels in the input image, to replace all with its median. The pixel values are then ordered in ascending order from smallest to largest. Choosing the in-between value for the new pixels to get the median is the final step^22^. Thus, the image’s texture is preserved through pre-processing.

The canny edge detection (CED) algorithm is implemented following adaptive median filtering. The median filtered image’s intensity gradient is initially identified. Following that is the non-maximum suppression to get rid of the fake result to border detection. Applying the threshold to identify potential borders is the next step. In the end, each weak edges that are not to strong edges are suppressed to locate edges. The CED selects a 2 × 2 neighbor area when estimating the image gradient, also when defining the direction and magnitude of the gradient. The first-order deviation of the image directions can then be constructed.

The weak edge as well as strong edge suppress the gradient value with values of 1 for strong edge, equal to zero, as well as 0 for weak edge. The current pixel edge strength along with border strength in the negative as well as positive gradient directions are then compared. When non-maximum suppression is used, edge pixels produce a false result. The value will remain intact if the pixel’s edge strength exceeds than other pixels^23^.

The tumor’s origin uses the threshold approach to transform the MRI scan into a binary image. Using the non-maximum suppression $\:Img\left[a,b\right]$ formula does not stop the noise from producing local maxima. The thresholds are set to high as well as low, to eliminate these. It catches strong and weak edges. Hysteresis thresholding is applied to low threshold (TL) as well as high threshold (TH) to the $\:Img\left[a,b\right]$ for calculating the edge gradient and needs two threshold values such as the maximum as well as smallest. In this instance, TL, TH, $\:Img\left[a,b\right]$ each point was scanned as well as fixed to locate the subsequent non-visited pixel.

1 $$\:Img\left[a,b\right]>TH$$

Starting from $\:Img\left[a,b\right]$, it follows the connected local maxima in both perpendicular directions as they approach the normal edges.

2 $$\:\:\:\:\:\:\:\:\:\:\:\:\:\:\:\:\:\:\:\:Img\left[a,b\right]>TL$$

Marking all visited points as well as compiling their positions list makes it easier to find the points in the connected contour. For adaptive median filtering, a 5 × 5 window size is selected after evaluating several configurations. For Canny edge detection, the thresholds are tuned experimentally as 0.05 for low threshold and 0.15 for high threshold. These values are optimized to ensure tumor edges and are preserved without introducing additional noise. Sensitivity analysis demonstrated that tighter thresholds enhance fine boundary preservation which is required for accurate classification. As a result, gray along with white matter regions of interest must be identified. The MRI image’s white matter and grey matter are both subjected to these calculated high and low thresholds. The output could be a list, with each item describing the image location of a related contour. There is a tendency to concentrate on the nearest pixels when comparing the edge value to the spreading space realization. Lastly, morphological procedures are used to highpoint the tumor on the MRI images in the affected area using segmentation. The stacked custom CNN is then used to classify the tumor from the filtered segmented image.

### Stacked custom convolutional neural network for imaging classification

In practice, the training convergence can be significantly speed up by initializing the network using a pretrained model, trained with large datasets. Additionally, it reduces overfitting and improves accuracy, making it necessary to train the entire network. For training, the network selects data from the previous section, resolving the classification problem. Although transformer architectures have recently shown strong performance in medical imaging tasks^2–6^, CNNs remain highly effective for voxel-based morphometric analysis where local spatial dependencies dominate. Transformers, while powerful, require significantly larger datasets to avoid overfitting and are computationally heavier for voxel-level tasks. Given the dataset size and the objective of capturing fine-grained brain tissue changes, CNNs offer a better balance between complexity, interpretability, and accuracy in this study. Vision Transformers (ViTs) have shown strong performance in medical imaging tasks, particularly when leveraging pre-trained models through transfer learning. Studies such as^24–26^ have demonstrated that transformer-based architectures can achieve high performance on large-scale non-medical data when fine-tuned on medical datasets, regardless of initial training. However, ViTs require important computational resources and often large datasets to avoid overfitting. As this study focuses on voxel-level classification using a comparably modest dataset, CNNs offer better control over spatial hierarchies and local feature learning with fewer parameters. Moreover, the custom activation function and enhanced VBM-based preprocessing are designed specifically for CNN frameworks, making a combination with ViTs non-trivial. Future work will include benchmarking against transformer-based backbones, but for the current scope, CNNs present a more interpretable and resource-efficient choice.

CNN maintains the spatial connection when processing high-dimensional arrays. Typical convolutional neural networks (CNNs) consist of a classification layer, which is responsible for classification, and a sequence of convolutional and pooling layers. This proposed stacked custom CNN has the following layers: an input layer, three batch normalization layers, three convolution2d layers, three custom layers (substituting the default layers), two pooling layers, a final classification layer, a fully connected layer, along with a softmax layer. The term “convolution” refers to the operation that these layers perform. The input filtered also morphology image can be scanned with this function to generate feature maps using a filter—a number matrix. Edges, for example, are one characteristic that each filter takes from the image. Training teaches how to use convolutional filters in the best way for the job at hand. In the convolutional layer, a learnable filters series are convolved to produce feature maps for all filters.

The batch normalization layer uses the standard deviation as well as mean of the mini-batch to equalize the inputs. The objective of the batch normalization layer is to stabilize the distribution of activation values during training. Pooling layers let the CNN associate data from convolutional layers, which reduces distortion invariance and dimensionality. The pooling layer is responsible for decreasing the feature maps spatial size. This helps to decrease the number of learnable parameters and the cost of computing, thereby reducing the number of over-fitting issues. The task of neurons linking to each activation is done by the fully connection layer. A probability score is produced by the soft-max layer for all distinct class. The classification layer then categorizes the various tumor classes. With more layers, the CNNs’ data representations become more complex.

The details of the custom layer which is created manually by using a class definition is described here. This layer replaces the default used activation layers. In the proposed network, the CNN activation processes are located in the hidden and output nodes. The activation functions convert the input value that comes to the node into a modified version before sending it on to the next generation of neurons. For activation functions, the derivative approaches 0 due to function saturation. This effectively cuts off the gradient’s ability to recirculate through the system. The learning algorithm has trouble continuing to change the weights because of this^27,28^. To get around this problem, the ReLU activation function returns 0 for negative inputs and 1 for positive ones. Here is the statement (3):

3 $$\:relu\left(u\right)={max}\left(u,0\right)$$

When training a model, the gradient may explode, but a ReLU with a maximum cap can handle this. Additionally, the vanishing gradient issue occurs due to heavy weights. The ‘dead’ neurons from the negative inputs are set to 0 by the usage of a standard RELU. Owing to the computational efficiency of the RELU, the network can converge relatively quickly. RELU is also nonlinear, despite the fact that it appears to be linear. It supports back propagation and has a derivative function. However, when inputs are negative or close to zero, ReLU encounters the dying problem. The gradient of the purpose is zero at this point, backpropagation is impossible for the network, and learning is also impossible. Because it allows backpropagation even for negative input values and has a slight positive slope in the negative region, the leaky ReLU activation function is recommended for avoiding the dying ReLU problem^29^. This is shown mathematically as in (4)

4 $$\:Lrelu\left(u\right)={max}\left(u,0.01u\right)$$

Inconsistent outcomes are a consequence of Leaky ReLU’s inability to reliably predict negative input values. Therefore, the selected parametric ReLU activation function, unlike leaky ReLU, utilizes the goal’s negative slope as a statement, thereby facilitating the learning of the negative slope. Consequently, backpropagation may discover the optimal value for k^29,30^. Here is the mathematical formula (5):

5 $$\:{\:Pr}elu\left(u\right)={max}\left(0,u\right)+k{min}\left(0,u\right)$$

However, PRELU may act differently based on the problem. An improved softsign activation is utilized in the work termed as SPR-eLu activation function. The proposed SPR-eLu activation function is formulated by combining the all ReLU, PReLU, and Softsign activations characteristics. As per it ReLU ensures sparse activations, PReLU handles negative values dynamically and Softsign provides smoother asymptotic behavior to enhance convergence. The mathematical formulation for proposed SPR-eLu activation function merges these advantages:

- Positive input: acts similar to capped ReLU.
- Negative input: allows a learnable slope as in PReLU but smoothens the transition like Softsign.

This is stated as follows as in (6):

6 $$\:softsign\left(u\right)=\frac{u}{\left|u\right|+1}$$

The data are compressed using the Softsign function into the range (−1, 1); and the center of the output is 0. The initialization technique is more stable, but the asymptote line is smoother and the saturation and range approach 0 more slowly. The large Softsign function center, the smaller region at u = 0, and the high level of nonlinearization enable an easy definition of the more challenging boundary. Located approximately in the center of the nonlinear section is the softsign function, which serves as a polynomial nonlinear activation purpose. Gentle saturation simplifies the computation, minimizes iterations, and facilitates easy convergence. All the values of every layer utilized is tabulated in Table 1.

**Table 1:** Layer configuration.

Layers	Values
Input Layer	Input size 240 × 320 × 1 (grayscale MRI)
Convolution Layer 1	32 filters, kernel size 3 × 3, stride 1, padding same, followed by Batch Normalization and custom SPR-eLu activation
Convolution Layer 2	64 filters, kernel size 3 × 3, Batch Normalization and custom SPR-eLu activation
Max-Pooling Layer	Pool size 2 × 2
Convolution Layer 3	128 filters, kernel size 3 × 3, Batch Normalization and custom SPR-eLu activation
Max-Pooling Layer	Pool size 2 × 2
Fully Connected Layer	512 neurons with SPR-eLu activation
Softmax Layer	Classifies into tumor/no-tumor
Classification Layer	Output final prediction

This work proposes a novel unsaturated segment neuron activation function, drawing on ReLU, PReLU, as well as the Softsign function features. When the data is more than zero, the ReLU governs the sparse ability. When the data is less than zero, the P-RELU and Softsign functions take into account the negative axis message and modify the data distribution for increased fault tolerance. We use a predict function in this custom activation function layer to advance the input data as well as output the outcome. Equation (7) demonstrates that this work’s predict function uses an improved activation function known as the SPR-eLu function.

7 $$\:SPR-elu\left(u\right)={max}\left(0,u\right)+{min}\left(0,a*\frac{u}{\left|u\right|+1}\right)$$

A backward function is also developed to transmit the loss function derivative backwards through the layer. The backward function is employed to propagate the loss function derivative across the layer. Also, this work considered an additional parameter termed as alpha, which is a parameter that can be learned from (−1,1).

**Fig. 2:**
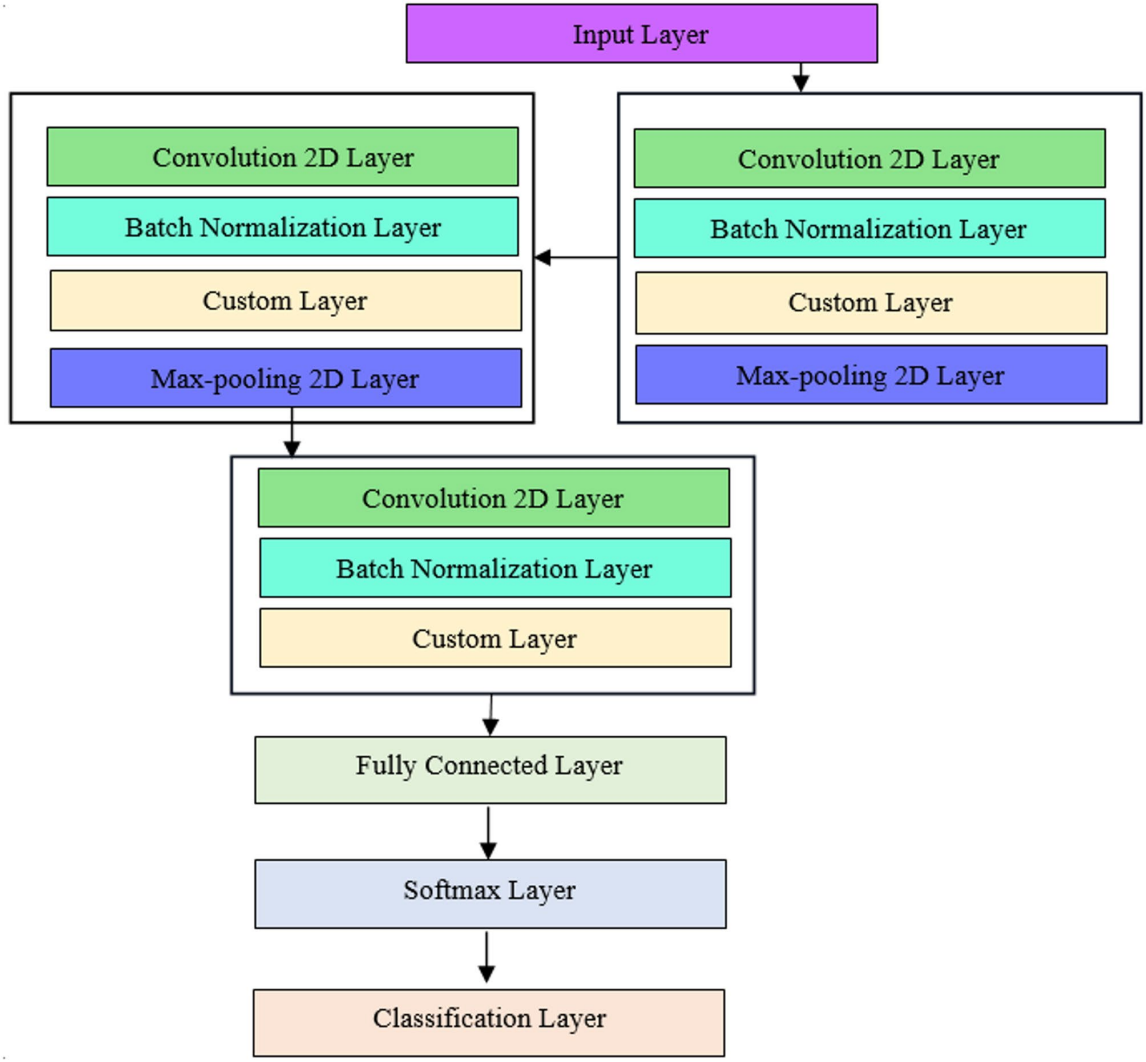
Architecture of proposed stacked custom CNN.

By employing this learnable parameter, a learnable parameter will be passed within the custom layer created in this work. Additionally, the “Stochastic Gradient Descent with Momentum (SGDM)” optimizer is one of the training options in addition to the “adam” optimizer. The executing surroundings is set by taking into account the “SGDM” optimizer. Use the “ExecutionEnvironment” and “parallel” options to run trainNetwork or a custom training loop in parallel. Use the Pool option to specify the number of workers and use batch to operate on the cluster. Figure 2 depicts the proposed stacked custom CNN layer’s description.

## Results

The proposed system is implemented in MATLAB R2019a using an i5 CPU without GPU acceleration. The average training time per epoch is approximately 5 min. The full model memory footprint is approximately 200 MB. While MATLAB provides a convenient prototyping environment, migrating the model to TensorFlow or PyTorch enables GPU acceleration, potentially reducing training time by 2–3× and optimizing inference speed for real-world clinical deployment. The MRI brain image dataset used in this study is^31^, and it contains images of brain tumors i.e., with and without tumors in two categories. With this dataset, voxel-based morphometric processes like the adaptive median filter preprocessing and canny edge detection are carried out.

Primarily, the input dataset undergoes data augmentation. Hence, we preprocess this enhanced dataset using a median filter. Filtering comes next, followed by the clever edge detection of VBM. Delivering the astute edge-detected image marks the final level of categorization. This dataset includes 155 images for the “yes” class along with 98 images for the “no” class. There are about 1,148 images for the “no” class also 1,118 images for the “yes” class in the dataset after data augmentation. This data augmentation is used in stages including filtering, canny edge detection, as well as finally, classification. The training stage uses 80% of this categorization data, while the testing stage uses 20%^32^.

**Table 2:** Hyperparameters.

Hyperparameters	Values
Optimizer	SGDM
Initial learning rate	0.01
L2 Regularization	0.0005
Mini-batch size	128
Epochs	5
Execution environment	Parallel CPU workers (MATLAB Parallel Computing Toolbox)

**Fig. 3:**
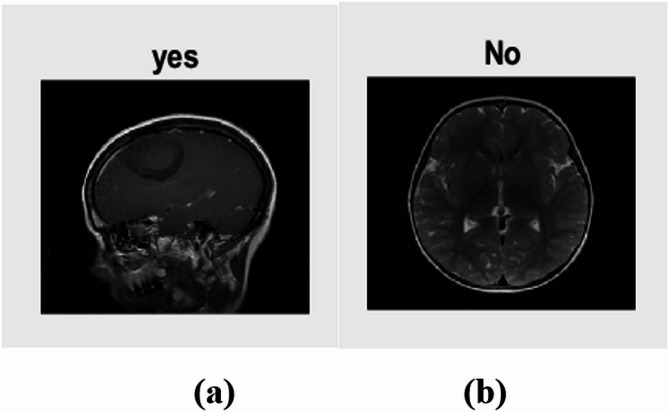
Input (**a**) With Tumor (**b**) Without tumor.

The proposed data augmentation trains a stacked custom CNN model with a batch size of 128 samples and an L2 regularization factor of 0.0005 over 5 epochs. They settled on the SGDM optimizer with a starting learning rate of 0.01. All the hyperparameters details are also described in Table 2. The hyperparameters are selected based on preliminary experiments and manual tuning to maximize validation accuracy while minimizing overfitting. In this work a tenfold cross-validation is used to eliminate performance bias in the enhanced, filtered, as well as segmented dataset. Figure 3 displays the brain images used in this study. The tumor is either present or absent in this case; “yes” indicates the former and “no” the latter.

### Dataset description

The MRI dataset used in this study is obtained from a public database on Kaggle (https://www.kaggle.com/datasets/navoneel/brain-mri-images-for-brain-tumor-detection ). The images were acquired using standard clinical MRI protocols with typical slice thickness and resolutions. In the dataset documentation, the acquisition parameters such as magnetic field strength and slice thickness were not provided. The dataset focuses on binary classification such as presence and absence of tumor. Preoperative images are included but no postoperative scans are present. The details about patient demographics such as age and gender are not available in this dataset documentation.

### Evaluation parameters

The parameters comprising specificity, negative prediction value (NPV), accuracy, precision, f1_score, is employed to evaluate the performance of the proposed VBM. The number of pixels correctly identified by the algorithm, also known as true positives, is known as sensitivity. Increasing the number of false positives (FPs) negatively impacts specificity, as it signifies the incorrect classification of more pixels as positive. The subsequent equations (8 to 13) mathematically represent all of these performance characteristics.

8 $$\:Sensitivity=\:\frac{TP}{FN+TP}$$

9 $$\:Specificity=\:\frac{TN}{FP+TN}$$

10 $$\:Accuracy=\:\frac{TN+TP}{FP+TP+FN+TN}$$

11 $$\:Precision=\:\frac{TP}{TP+FP}$$

12 $$\:F1\_score=\:2.\frac{precision*sensitivity}{precision+sensitivity}$$

13 $$\:negative\:predictive\:value=\:\frac{TN}{TN+FN}$$

where TP, TN, FP, and FN denote true negative, true positive, false negative, as well as false positive respectively. In the next section, the proposed work outcomes are given along with some existing works comparison.

### Comparative analysis

In this section, the proposed work is compared with other existing results which are also implemented with the same dataset. The existing methods compared included implementation done with aug with sharpening, aug with smoothening, aug with filtering plus smoothening as well as canny, aug with median filter and aug with filtering plus sharpening as well as canny. Time, validation loss, mini batch loss, as well as mini batch accuracy are the parameters compared within this Table 3.

**Table 3:** Comparison table of existing methods along with proposed algorithm Approaches.

Methods	Cross-validation	Epoch	Time (hh: mm: ss)	Mini-batch Accuracy (%)	Validation Accuracy (%)	Mini-batch Loss	Validation Loss	Base Learning Rate
Aug + median filter	1	5	00:09:28	97.66	99.52	0.0935	0.0447	0.0001
	2	5	00:09:18	98.44	98.00	0.0691	0.0677	0.0001
	3	5	00:08:44	97.66	98.81	0.0726	0.0544	0.0001
	4	5	00:08:47	96.88	98.81	0.0641	0.0547	0.0001
	5	5	00:08:34	100.00	99.57	0.0343	0.0350	0.0001
	6	5	00:07:05	95.31	98.24	0.1502	0.0636	0.0001
	7	5	00:05:54	99.22	99.24	0.0420	0.0522	0.0001
	8	5	00:04:50	97.66	98.86	0.0725	0.0530	0.0001
	9	5	00:05:16	100.00	99.52	0.0328	0.0370	0.0001
	10	5	00:05:26	100.00	99.43	0.0195	0.0383	0.0001
			00:07:20	98.28	99	0.0650	0.0500	0.0001
Aug + sharpening	1	5	00:09:39	100.00	99.76	0.0315	0.0386	0.0001
	2	5	00:12:39	96.09	98.57	0.0840	0.0625	0.0001
	3	5	00:11:24	97.66	98.62	0.1117	0.0703	0.0001
	4	5	00:11:23	100.00	98.62	0.0340	0.0621	0.0001
	5	5	00:10:16	100.00	99.57	0.0407	0.0413	0.0001
	6	5	00:09:14	96.88	98.71	0.0869	0.0741	0.0001
	7	5	00:09:14	96.09	99.14	0.1304	0.0579	0.0001
	8	5	00:10:32	98.44	99.00	0.0769	0.0677	0.0001
	9	5	00:10:40	99.22	99.67	0.0357	0.0332	0.0001
	10	5	00:07:29	96.88	99.38	0.1032	0.0510	0.0001
			00:10:15	98.12	99.10	0.0735	0.0558	0.0001
Aug + smoothening	1	5	00:06:32	100.00	99.28	0.0385	0.0465	0.0001
	2	5	00:07:34	97.66	99.14	0.0605	0.0458	0.0001
	3	5	00:07:19	98.44	98.28	0.1160	0.0692	0.0001
	4	5	00:07:21	96.09	95.33	0.0815	0.1068	0.0001
	5	5	00:05:27	99.22	99.09	0.0559	0.0421	0.0001
	6	5	00:06:53	96.88	97.95	0.0871	0.0812	0.0001
	7	5	00:07:51	97.66	98.24	0.1694	0.0687	0.0001
	8	5	00:08:48	96.09	98.71	0.0794	0.0565	0.0001
	9	5	00:09:02	97.66	98.86	0.0495	0.0430	0.0001
	10	5	00:08:59	96.88	99.38	0.0675	0.0348	0.0001
			00:07:35	97.65	98.42	0.0805	0.0594	0.0001
Aug + filter + smoothening + canny	1	5	00:04:41	99.22	99.19	0.0649	0.0695	0.0001
	2	5	00:05:12	100.00	99.71	0.0541	0.0528	0.0001
	3	5	00:05:18	97.66	99.09	0.0717	0.0792	0.0001
	4	5	00:05:27	100.00	99.62	0.0559	0.0515	0.0001
	5	5	00:05:11	98.44	99.48	0.0468	0.0339	0.0001
	6	5	00:05:10	96.88	98.57	0.1188	0.0844	0.0001
	7	5	00:05:17	97.66	98.24	0.0989	0.0911	0.0001
	8	5	00:05:21	99.22	99.81	0.0663	0.0529	0.0001
	9	5	00:05:36	99.22	99.76	0.0583	0.0465	0.0001
	10	5	00:05:23	100.00	99.67	0.0450	0.0487	0.0001
			00:05:16	98.83	99.31	0.0680	0.0610	0.0001
Aug + filter + sharpening + canny	1	5	00:09:21	97.66	98.62	0.0753	0.0766	0.0001
	2	5	00:08:51	96.09	98.38	0.1322	0.0860	0.0001
	3	5	00:08:35	96.88	99.00	0.1237	0.0743	0.0001
	4	5	00:08:46	94.53	98.24	0.1358	0.1017	0.0001
	5	5	00:08:15	99.22	99.43	0.0715	0.0484	0.0001
	6	5	00:08:22	96.88	99.14	0.1118	0.0628	0.0001
	7	5	00:05:30	98.44	99.33	0.0715	0.0649	0.0001
	8	5	00:06:12	99.22	98.95	0.0828	0.0770	0.0001
	9	5	00:05:39	100.00	99.28	0.0489	0.0503	0.0001
	10	5	00:07:17	99.22	99.00	0.0739	0.0759	0.0001
			00:07:41	97.81	98.93	0.0927	0.0717	0.0001
Proposed	1	5	00:04:26	97.66	99.81	0.0391	0.0200	0.0100
	2	5	00:04.33	96.88	99.24	0.0736	0.0334	0.0100
	3	5	00:05:12	92.19	94.75	0.1789	0.1148	0.0100
	4	5	00:04:50	98.44	99.24	0.0674	0.0410	0.0100
	5	5	00:04:31	98.44	99.09	0.0515	0.0303	0.0100
	6	5	00:04:59	95.31	99.28	0.0949	0.0376	0.0100
	7	5	00:05:31	97.66	98.86	0.0719	0.0411	0.0100
	8	5	00:05:02	96.88	99.24	0.0825	0.0374	0.0100
	9	5	00:04:39	100	99.90	0.0279	0.0132	0.0100
	10	5	00:04:40	98.44	98.52	0.0774	0.0549	0.0100
			00:04:23	99.17	99.67	0.0489	0.0423	0.0100

The algorithm requires a total time of 4 min and 23 s, with variations. The algorithm requires 7 min and 50 s for the analysis alone, 7 min and 20 s for the proposed filtering, 10 min and 15 s for the analysis with sharpening, 7 min and 35 s for the analysis with smoothing, 5 min and 16 s for the analysis with filtering, smoothing, and canny, and 7 min and 41 s for the analysis with additional filtering, and sharpening. Execution time for the proposed job is obviously lower. Consequently, the use of the parallel execution environment has significantly lowered the training time.

The proposed approach achieves an average mini-batch accuracy of 99.17%. Therefore, the proposed model is 0.90% better than Aug. with filtering, 1.07% better than Aug. with sharpening, 1.55% better than Aug. with filtering, 0.34% better than Aug. with filtering plus smoothing plus Canny, and 1.39% better than Aug. with filtering plus sharpening. The proposed model clearly outperforms the other existing algorithms in terms of accuracy. The proposed approach has an average validation accuracy of 99.67%. To compare, the proposed method outperforms aug with filtering by 0.41%, aug with sharpening by 0.31%, aug with smoothing by 1%, aug with filtering plus smoothing and canny by 0.10%, and aug with filtering plus sharpening plus canny by 0.10%. The proposed approach clearly outperforms the other existing algorithms in terms of accuracy.

The proposed model achieves an average mini-batch loss of 0.0489. When compared the proposed algorithm improves performance by 2.24% when aug combined with filtering, by 50.30% when combined with sharpening, by 64.62% when combined with smoothing, by 39.05% when combined with filtering, smoothing, and Canny, and by 89.57% when combined with sharpening and filtering. The proposed model has an average validation loss of 0.0423. When compared, the proposed model outperforms aug with filtering by 18.20%, sharpening by 29.55%, smoothing by 40.42%, filtering plus smoothing plus canny by 44.20%, and sharpening plus canny by 69.50%. As a result, it is clear that the proposed method exhibits a lower error rate than other existing algorithms.

**Fig. 4:**
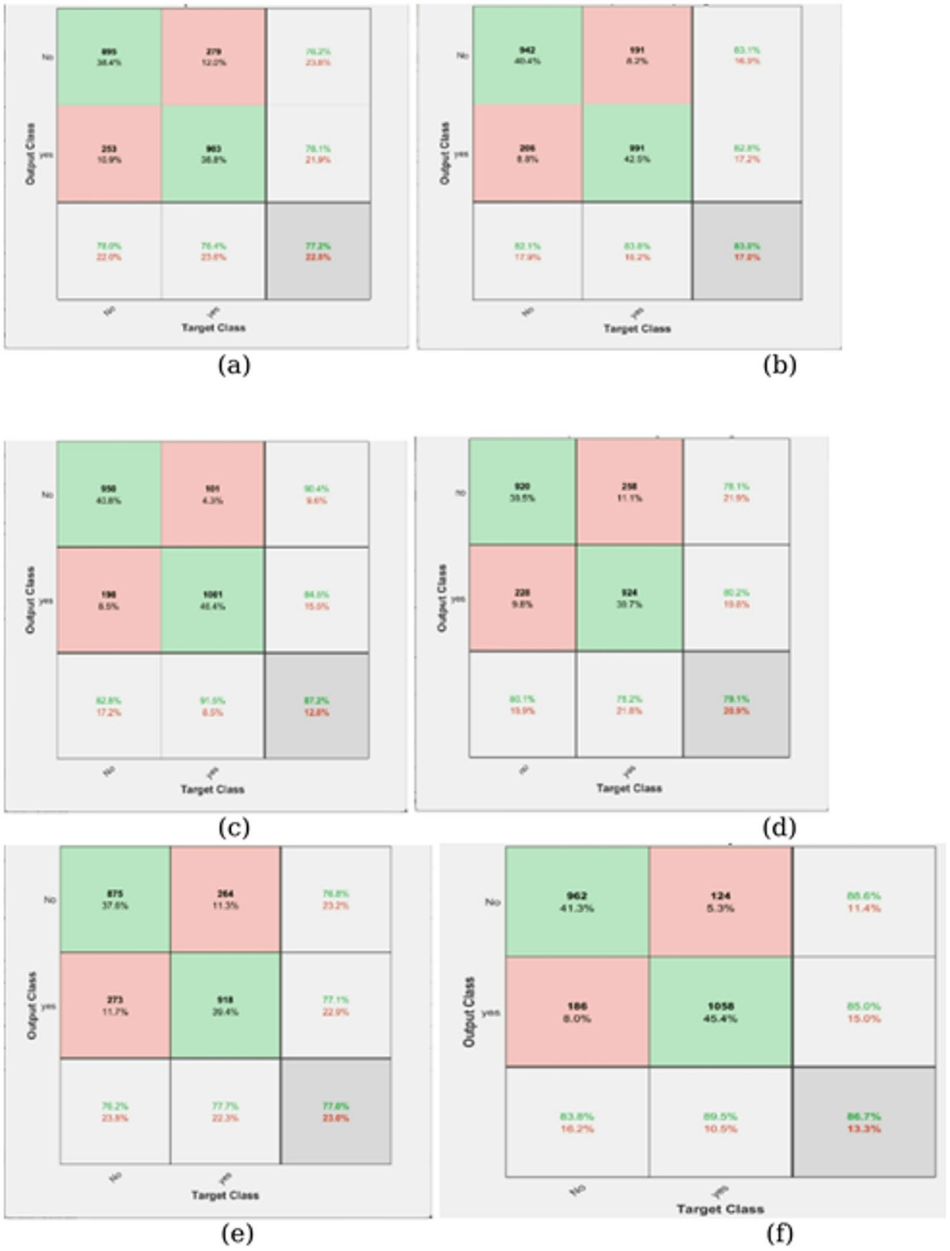
Confusion Matrix Graph (**a**) ICNN with data augmentation and filtering (**b**) ICNN with data augmentation and sharpening (**c**) ICNN with data augmentation and smoothing (**d**) ICNN with data augmentation + filtering + smoothing + canny (**e**) ICNN with data augmentation + filtering + sharpening + canny (**f**) Proposed Model.

**Fig. 5:**
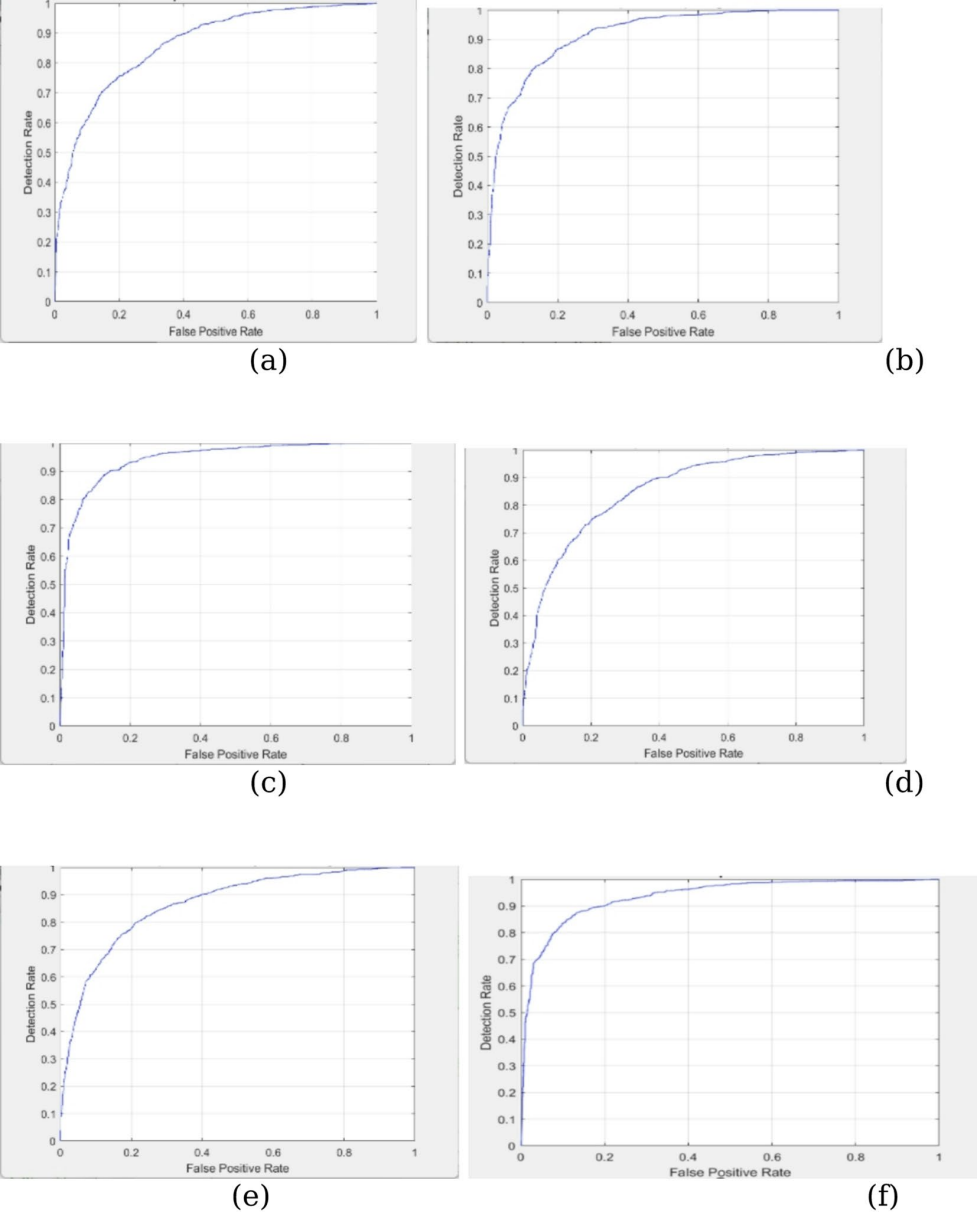
ROC Curve (**a**) ICNN with data augmentation and filtering (**b**) ICNN with data augmentation and sharpening (**c**) ICNN with data augmentation and smoothing (**d**) ICNN with data augmentation + filtering + smoothing + canny (**e**) ICNN with data augmentation + filtering + sharpening + canny (**f**) Proposed Model.

In Fig. 4, the proposed algorithm’s Confusion Matrix plot is shown alongside those of other existing methods. The ROC curve indicates the effectiveness of the proposed classification method. Furthermore, the estimate of the AUC (area under the ROC curve) is provided. The confusion matrix reveals that although the proposed model achieves high accuracy, occasional false negatives and false positives still occur. False negatives, where a tumor is misclassified as non-tumor, are clinically critical, as they could delay necessary treatment. The model’s false negative rate was low; hence, future refinements should focus particularly on minimizing such errors. Figure 5 shows the proposed algorithm’s ROC curve diagram together with those of other existing methods.

**Table 4:** Performance comparison for different activations.

Activation	Accuracy (%)	Loss stability	Convergence speed
ReLU	97.85	Medium	Medium
Leaky ReLU	98.15	Medium	Medium
SPR-eLu (Proposed)	99.17	High	Faster

From Table 4 it is evident than the SPR-eLu activation increased training convergence, improved generalization, and reduced loss oscillations compared to standard ReLU-based models. Table 5 presents comparisons with recent state-of-the-art methods as reported in the literature, to further contextualize the results.

**Table 5:** Performance comparison with recent Methods.

Method	Accuracy (%)	AUC
Swin Transformer [5]	98.2	0.960
U-KAN [21]	98.5	0.965
P2SAM [23]	97.9	0.955
Proposed Stacked Custom CNN	99.17	0.966

The proposed method demonstrates better performance, while maintaining lower computational complexity when compared to existing transformer-based methods.

**Table 6:** Performance comparison table.

Method	Classes	Sensitivity	Specificity	Precision	NPV	F1-score	AUC
Proposed	0	0.8653	0.9089	0.9014	0.8765	0.8986	0.9660
	1	0.9078	0.8587	0.8689	0.8765	0.8889	
	1	0.9112	0.8432	0.8568	0.9021	0.8831	
Aug + median filter	0	0.7796	0.7640	0.7624	0.7811	0.7709	0.8613
	1	0.7640	0.7796	0.7811	0.7624	0.7725	
Aug + sharpening	0	0.8206	0.8384	0.8314	0.8279	0.8260	0.9152
	1	0.8384	0.8206	0.8279	0.8314	0.8331	
Aug + smoothening	0	0.8275	0.9146	0.9039	0.8452	0.8640	0.9447
	1	0.9146	0.8275	0.8452	0.9039	0.8785	
Aug + filter + smoothening + canny	0	0.8014	0.7817	0.7810	0.8021	0.7911	0.8669
	1	0.7817	0.8014	0.8021	0.7810	0.7918	
Aug + filter + sharpening + canny	0	0.7622	0.7766	0.7682	0.7708	0.7652	0.8546
	1	0.7766	0.7622	0.7708	0.7682	0.7737	

Table 6 compares the performance of the proposed with the other exiting algorithms implemented in terms of the parameters including precision, NPV, F1-score, sensitivity, specificity as well as AUC value. The results for both the classes are given. It is noticed from the table that the proposed algorithm shows improved results for every parameter. This is because the proposed algorithm uses augmentation, smoothening, median filter, canny edge detection along with the use of stacked CNN layers. By augmentation the number of images is increased and this is an initial step to increase the performance of the proposed model. Since in DL algorithms the performance will increase with increment in number of images. Then in proposed rather than the sharpening operation smoothening operation is only considered. Since when the results for the smoothening results are only better. Then median filter is also used which is responsible for elimination of any noise in input image. Thus, this filter process also helps in improving the classification performance. Then again, a canny edge detection algorithm is employed to edges in the image. Finally, the proposed stacked CNN algorithm is responsible to provide the better classification performance. Though better results are provided with the proposed algorithm it has some less performance when checking with loss. So, minimizing this loss further will be considered as a future work. From Table 6 it is obvious that the proposed algorithm displays improved performance comparing with the existing algorithms. Finally, the presence and absence of tumor is detected correctly.

## Conclusion

Brain tumors, whether benign or malignant, symbolize a significant clinical challenge owing to their potential to cause severe neurological impairment or fatal outcomes through compression of critical brain structures. For effective plan of treatment and prognosis, accurate early diagnosis and classification are important. Using an improved voxel-based morphological technique on brain MRI images, this research was able to identify and classify the tumor. Using layered custom CNNs and image methods, the deployed system tries to classify brain images. Initially, preprocessing is done with the raw images through normalization, followed by threshold-based segmentation. Consequently, a stacked custom CNN model is done, that takes labeled segmentation information, to classify brain MRI images. The findings accurately categorize the top tumor classifications with a precision of 99.67%. Additionally, some restrictions must be considered. This study implements strategies to further minimize future errors, as the reduction in loss remains unaltered. However, although the experiment did prove that the DL network’s learned information is transferable to other parts of the body, it only did so in the brain. Therefore, we will implement strategies to collaborate with diverse datasets in the future. Subsequent rounds will also further optimize the model. The proposed system has the ability to serve as a decision-support tool in clinical settings. It can be integrated into Picture Archiving and Communication Systems (PACS) to aid radiologists during preliminary screenings or deployed in multidisciplinary team (MDT) meetings to support treatment discussions. Future developments will include real-time inference capabilities and prospective clinical trials to validate its utility within clinical workflows. A present study limitation is the absence of modeling sequential dependencies across MRI slices. Ignoring inter-slice spatial correlations restricts model performance, since MRI images are typically acquired as sequential slices for a single patient. Hence, future work will explore 3D CNNs or sequential models like recurrent architectures or transformers to capture these dependencies. Future work will incorporate multi-type datasets with clinical metadata to enhance interpretability.

## Data Availability

[https://www.kaggle.com/datasets/navoneel/brain-mri-images-for-brain-tumor-detection ](https:/www.kaggle.com/datasets/navoneel/brain-mri-images-for-brain-tumor-detection ).
